# Hypothesis-generating clinical genomics research and predictive medicine

**DOI:** 10.1186/1753-6561-6-S6-O6

**Published:** 2012-10-01

**Authors:** Leslie Biesecker

**Affiliations:** 1 National Human Genome Research Institute, USA

## 

The advent of affordable genome and exome sequencing provides incredible opportunities and poses significant challenges for clinical research and clinical care. For the first time, it is technically feasible to access the entire genetic architecture of a phenotype. The dissection of this genetic architecture of disease will yield unprecedented insights into molecular pathophysiology and provide numerous therapeutic targets. Soon, the primary etiology of all Mendelian traits will be elucidated and modifiers will follow. Translating this genetic architecture into diagnostics and therapeutics will be feasible and will require creative, aggressive and thoughtful approaches to numerous challenges. One of the first applications will be predictive medicine, which should initially focus on high-penetrance Mendelian phenocopies of common diseases and disorders with effective interventions. These include cancer susceptibility syndromes, cardiomyopathies and dysrhythmias, malignant hyperthermia, dyslipidemias, and a host of other disorders. These approaches will require improved abilities to predict phenotype from genotype and a clinical paradigm shift that supports a disease screening approach (as distinct from a differential diagnosis approach). As well, researcher and clinicians will need to develop creative approaches to dealing with data overload - no physician (research or clinical) can address three million variations. Prioritizing these variants into clinically appropriate categories is urgent, as is developing an unbiased assessment of penetrance in variable expressivity. I will give examples of several of these approaches and their attendant challenges from the ClinSeq project in the intramural NIH.

